# PAH clearance after renal ischemia and reperfusion is a function of impaired expression of basolateral Oat1 and Oat3

**DOI:** 10.1002/phy2.243

**Published:** 2014-02-19

**Authors:** Ariane Bischoff, Michael Bucher, Michael Gekle, Christoph Sauvant

**Affiliations:** 1 Klinik für Anästhesie und Operative Intensivmedizin, Universitätsklinikum Halle, Halle (Saale), Germany; 2 Julius‐Bernstein‐Institut für Physiologie, Universität Halle‐Wittenberg, Halle (Saale), Germany

**Keywords:** clearance, experimental, ischemic acute kidney injury, perfusion

## Abstract

Determination of renal plasma flow (RPF) by para‐aminohippurate (PAH) clearance leads to gross underestimation of this respective parameter due to impaired renal extraction of PAH after renal ischemia and reperfusion injury. However, no mechanistic explanation for this phenomenon is available. Based on our own previous studies we hypothesized that this may be due to impairment of expression of the basolateral rate limiting organic anion transporters Oat1 and Oat3. Thus, we investigated this phenomenon in a rat model of renal ischemia and reperfusion by determining PAH clearance, PAH extraction, PAH net secretion, and the expression of rOat1 and rOat3. PAH extraction was seriously impaired after ischemia and reperfusion which led to a threefold underestimation of RPF when PAH extraction ratio was not considered. PAH extraction directly correlated with the expression of basolateral Oat1 and Oat3. Tubular PAH secretion directly correlated with PAH extraction. Consequently, our data offer an explanation for impaired renal PAH extraction by reduced expression of the rate limiting basolateral organic anion transporters Oat1 and Oat3. Moreover, we show that determination of PAH net secretion is suitable to correct PAH clearance for impaired extraction after ischemia and reperfusion in order to get valid results for RPF.

## Abstract (precis)

Determination of renal plasma flow by PAH clearance leads to gross underestimation of this respective parameter due to impaired renal extraction of PAH after renal ischemia and reperfusion injury. However, no mechanistic explanation for this phenomenon was available up to now. Consequently, our data offer an explanation for impaired renal PAH extraction by reduced expression of the rate limiting basolateral organic anion transporters Oat1 and Oat3.

## Introduction

It has been shown that in human renal allograft the clearance of the prototypical organic anion para‐aminohippurate (PAH) was reduced for at least 7 days after transplantation (Corrigan et al. 1999). Based on the latter observation, we performed a study showing down regulation of the rate limiting basolateral organic anion transporters Oat1 and Oat3 during reperfusion after ischemic acute kidney injury (iAKI) (Schneider et al. 2007), which was confirmed by independent groups (Matsuzaki et al. 2007; Di Giusto et al. 2008).

Oat1 and Oat3 represent the classical renal basolateral polyspecific uptake transporters for organic anions (Dantzler and Wright 2003; Sekine et al. 2006; Rizwan and Burckhardt 2007) and are part of the proximal tubular transport system which secretes organic anions. This system consists of the mentioned organic anion exchangers located at the basolateral membrane (Oat1 and Oat3) representing the rate determining step of elimination and the efflux step at the apical membrane (Dantzler 2002; El‐Sheikh et al. 2008). The organic anion transport system of the renal proximal tubule plays a crucial role in the excretion of a variety of drugs or potentially toxic compounds (Perri et al. 2003; Van Montfoort et al. 2003).

Physiologically, this system extracts and secretes organic anions almost completely. Consequently, PAH extraction ratio in healthy humans was shown to approximate 0.9 (Warren et al. 1944; Battilana et al. 1991) (and it was determined as 0.82 ± 0.05 in sham operated control rats in our study). Smith proposed the clearance of the prototypical substrate PAH as a measure for renal blood flow already in 1938 (Smith et al. 1938). However, PAH clearance is a valid measure for renal blood flow only if (1) renal extraction of PAH is almost complete and if (2) renal extraction is not changed due to experimental conditions. Extraction corrected PAH clearance (corPAH clearance) is the more authentic measure for determination of renal blood flow as (1) it truly determines renal extraction of PAH in every animal investigated which makes it a reliable measure even to compare animals subjected to different experimental conditions that (2) may have varying PAH extraction ratios.

In the above mentioned study from Corrigan et al. (1999), the authors concluded that the renal perfusion as determined by PAH clearance after renal ischemia and reperfusion is highly underestimated due to impaired PAH extraction. In their study, however, PAH extraction was directly determined exclusively after 1–3 h after reperfusion of the graft kidneys whereas PAH extraction was only estimated in the reperfusion phase (7 days after ischemia).

**Figure 1.**
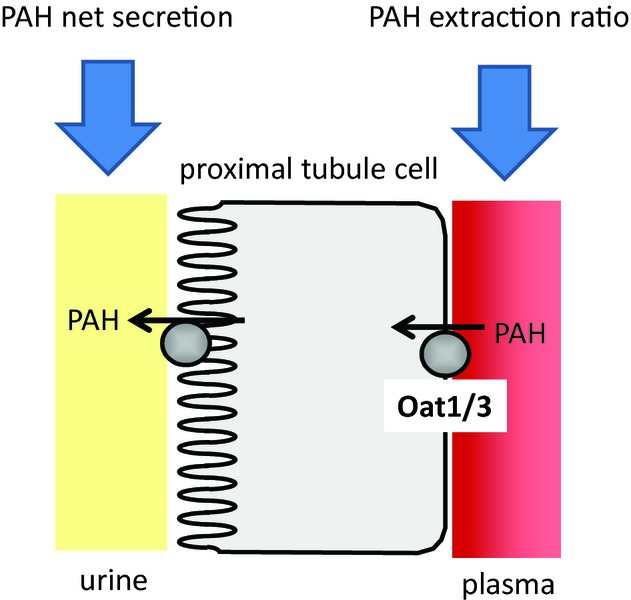
Cartoon illustrating para‐aminohippurate (PAH) net secretion and PAH excretion ratio. The equations for calculation of the respective parameters are given in the methods section.

In former studies we have shown that the proximal tubular secretion of PAH (determined as PAH net secretion; PNS) is reduced after renal ischemia and reperfusion which is accompanied by impaired expression of Oat1 and Oat3 (Schneider et al. 2007, 2009). In principle, however, PNS is a measure for transepithelial secretion of PAH, as it is determined with parameters detected in urine and thus maps total transcellular secretory transport of PAH into the tubular lumen (Fig. 1) which is dependent on the apical efflux step *and* basolateral uptake. The parameter reflecting basolateral uptake of PAH into the proximal tubule cells and therefore expression of rOat1/3 is the renal extraction of PAH from renal plasma (Fig. 1; equation given in the methods section). Moreover, this parameter allows correction of PAH clearance in order to obtain a true measure for renal perfusion (corPAH clearance; equation given in the methods section). (In fact impairment of luminal exit step would also indirectly impair basolateral uptake as saturation of the intracellular space will occur rapidly.)

In the present study, we wanted to determine the true renal plasma flow (RPF) after ischemia and reperfusion in rats and moreover gain evidence whether impaired expression of the rate limiting organic anion transporters rOat1 and rOat3 is sufficient to explain the decrease in PAH extraction after renal ischemia and reperfusion in our well‐established rat model system. Moreover, we tested whether the above mentioned impairment of expression explains gross underestimation of RPF during reperfusion after ischemia.

## Materials and Methods

### In vivo experimental procedure

Experiments were performed as published recently, (Schneider et al. 2007, 2009) where I/R injury was induced by bilateral clamping of renal arteries for 45 min in rats. Female Sprague‐Dawley rats (200–250 g body weight) were obtained from Charles River (Kissleg, Germany). After a period of at least 24 h in cages within a temperature‐controlled room with 14 h light‐and 10 h dark cycle and standard food with free access to tap water, anesthesia was performed by intraperitoneal application of xylacin hydrochloride (10 mg/kg body weight) and ketamine (100 mg/kg body weight). All operative procedures were performed on thermoregulated heating boards to maintain body temperature at 37.0°C. Postoperative pain relief was assured by subcutaneously administered tramadol (0.05 mg/kg body weight) and postoperative dehydration was prevented by subcutaneous administration of additional 1.0 mL 0.9% NaCl. Animals were divided into the following subgroups: Sham group (sham‐operation and supplementation with saline) or Clamping group (bilateral clamping and supplementation with saline). The care of animals and experimental procedures performed in this study were in accordance with the German law for animal protection and in accordance with the guidelines from the ethics review committee of the Bundesland Saxony‐Anhalt according to the approval 42502‐2‐1066MLU.

### Parameters of renal function and organic anion handling

Inulin‐ and PAH‐clearances were determined as described recently (Schneider et al. 2007, 2009). In addition to what is described therein, the renal vein was cannulized in order to gain plasma right after its passage through the kidney in order to calculate renal PAH extraction ratio as described below.

Calculations of inulin clearance and PAH clearance, PAH net secretion (PNS), renal extraction of PAH (E_PAH_), and PAH clearance corrected for renal PAH extraction (corPAH clearance) were performed according to the equations:

inulin clearance = (*I*_U_ × *V*_U_)/(*I*_A_ × t); in mL/min

PAH clearance = (PAH_U_ × *V*_U_)/(PAH_A_ × t); in mL/min

PNS = [(PAH_U_ × *V*_U_)/t] – [GFR × PAH_A_]; in *μ*g/min

E_PAH_ = (PAH_A_ – PAH_RV_)/PAH_A_; as fraction

corPAH clearance = [*V*_U_ × (PAH_U_ – PAH_RV_)]/[(PAH_A_ – PAH_RV_) × t]; in mL/min

where *I*_U_ is inulin concentration in urine; PAH_U_ is PAH concentration in urine; *I*_A_ is inulin concentration in arterial plasma; PAH_A_ is PAH concentration in arterial plasma; PAH_RV_ is the PAH concentration in plasma from the renal vein and *V*_U_ is urine volume.

### Realtime‐R(everse)T(ranscriptase)‐PCR

In brief, RT‐PCR was performed according to iQ SYBR‐Green Supermix RT‐PCR system protocol (Biorad, CA, USA). PCR amplification protocol and primers were used as recently described (Sauvant et al. 2009, 2010). Quantification was performed using the ΔΔC_T_ method using ß‐actin as reference gene and expression in control cells was normalized to 1.

### Data analysis

Data are presented as mean ± SEM. The *n* value is given in the text or in the figures. For all experiments, *n* equals the number of rats or the number of experiments (RT‐PCR, Western‐Blot) with tissue or tissue extractions from distinctive rats. Statistical significance was determined by unpaired Student's *t*‐test or ANOVA as appropriate. Data from clamped animals were tested against sham operated animals. Differences were considered statistically significant when *P *<**0.05.

## Results and Discussion

**Figure 2.**
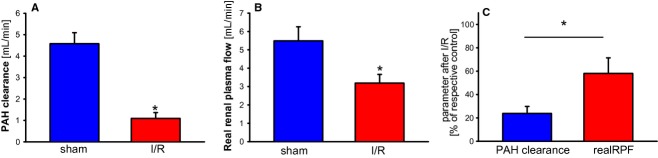
(A) para‐aminohippurate (PAH) clearance in rats 24 h after renal ischemia. PAH clearance was determined as given in the methods section. Rats were either sham operated or subjected to renal ischemia. *indicates statistical significant difference from sham operated animals. *n* = 8 for the respective experimental groups. (B) Real renal plasma flow (RPF) in rats 24 h after renal ischemia. Real RPF was calculated as corrected PAH clearance (corrPAH clearance) as given in the methods section. Rats were either sham operated or subjected to renal ischemia. *indicates statistical significant difference from sham operated animals. *n* = 8 for the respective experimental groups. (C) Comparison of PAH clearance and Real RPF in rats 24 h after renal ischemia. PAH clearance and Real RPF was calculated as corrected PAH clearance (corrPAH clearance) as given in the methods section. The respective mean in sham treated animals was set as 100% and the respective parameters in the animals subjected to ischemia and reperfusion were calculated in% in order to allow statistical comparison. Rats were either sham operated or subjected to renal ischemia. *indicates statistical significant difference from sham operated animals. *n* = 8 for the respective experimental groups.

As compared to PAH clearance in sham treated control animals, PAH clearance in animals subjected to ischemia was tremendously decreased after 24 h reperfusion (Fig. 2A). The impairment of PAH clearance is in the range of our own data published before (Schneider et al. 2009) indicating the reproducibility and reliability of the particular ischemia and reperfusion model used. The same is true for glomerular filtration rate (GFR) determined by inulin clearance which declines down to around 20% 24 h after ischemia as compared to the sham treated animals.

We have described before that the expression of the rate limiting basolateral organic anion transporters of the proximal tubule Oat1 and Oat3 is reduced during reperfusion after renal ischemia in rats (Schneider et al. 2007, 2009). Thus, it seemed obvious that PAH clearance is strongly influenced by the latter phenomenon due to a reduction of PAH extraction ratio which reflects basolateral uptake of organic anions into the proximal tubules. Consequently, we found that PAH clearance corrected for PAH extraction is only reduced to around 60% of the values in sham operated animals (Fig. 2B) as compared to a reduction down to 20% when PAH extraction is not considered (Fig. 2A). Thus, if PAH clearance is not corrected for renal PAH extraction (Fig. 2C), the impairment of renal perfusion caused by renal ischemia and reperfusion damage is threefold overestimated. As a consequence, decline of filtration fraction (GFR/RPF) is underestimated by the factor of 3 if RPF is determined by PAH clearance which is not corrected for PAH extraction. Noteworthy, these data are in good agreement with the observations in human renal allographs suffering from acute renal failure by Corrigan et al. (1999). In this particular study PAH clearance (in mL × min^−1^ × m^−2^) went down from 100% (340 ± 66) in healthy controls to 15% (50 ± 48) in transplanted kidneys after renal ischemia and reperfusion, whereas corrected PAH clearance (or true RPF as referred by Corrigan) went down from 100% (347 ± 67) to 58% (202 ± 72). Thus, impairment of renal perfusion during reperfusion after ischemia is highly overestimated when PAH extraction is not considered for determination of PAH clearance.

**Figure 3.**
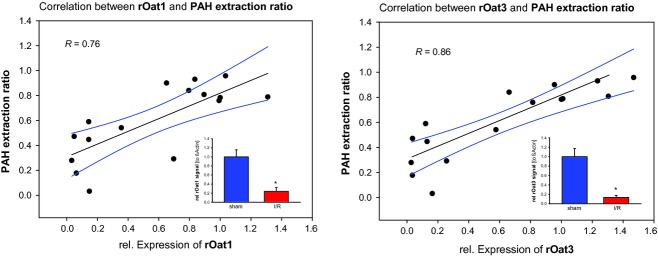
(A) Correlation of para‐aminohippurate (PAH) extraction ratio to relative expression of Oat1. Parameters were determined and calculated as given in the methods section and were grouped for every single animal (*n* = 16). Data were obtained from rats that were either sham operated or subjected to renal ischemia. The inlay is showing relative expression of Oat1 determined by qPCR *indicates statistical significant difference from sham operated animals. *n* = 8 for the respective experimental groups. (B) Correlation of PAH extraction ratio to relative expression of Oat3. Parameters were determined and calculated as given in the methods section and were grouped for every single animal (*n* = 16). Data were obtained from rats that were either sham operated or subjected to renal ischemia. The inlay is showing relative expression of Oat1 determined by qPCR *indicates statistical significant difference from sham operated animals. *n* = 8 for the respective experimental groups.

As we hypothesized that impairment of Oat expression is causal for reduced PAH extraction ratio during reperfusion after renal ischemia we determined the expression of Oat1 and Oat3 by qPCR. As shown before for Oat1 and Oat3 during reperfusion after ischemia the amount of protein behaves according to its respective mRNA (Schneider et al. 2007, 2009). Therefore, we correlated qPCR expression data with the respective PAH extraction ratio for each animal. As shown in Fig. 3A and B, there is a good overall correlation between expression of Oat1 or Oat3 and PAH extraction ratio. Thus, expression of Oat1 and Oat3 determines PAH extraction ratio. We conclude that the extraction ratio of PAH during reperfusion after renal ischemia is impaired due to reduced expression of Oat1 and Oat3. This will also be true for any other organic anion which is prone to renal elimination [for overview about drugs as substrates refer to Rizwan and Burckhardt (2007)].

**Figure 4.**
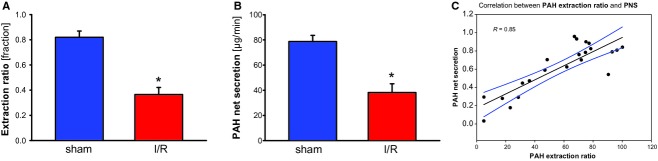
(A) para‐aminohippurate (PAH) extraction ratio in rats 24 h after renal ischemia. PAH extraction ratio was determined as given in the methods section. Rats were either sham operated or subjected to renal ischemia. *indicates statistical significant difference from sham operated animals. *n* = 10 for the respective experimental groups. (B) PAH net secretion in rats 24 h after renal ischemia. PAH net secretion was determined as given in the methods section. Rats were either sham operated or subjected to renal ischemia. *indicates statistical significant difference from sham operated animals. *n* = 10 for the respective experimental groups. (C) Correlation of PAH net secretion to PAH extraction ratio. Parameters were determined and calculated as given in the methods section and were grouped for every single animal (*n* = 16). Data were obtained from rats that were either sham operated or subjected to renal ischemia.

PAH extraction (ratio) is reflecting basolateral uptake of organic anions into the proximal tubule epithelial cell, which is significantly impaired during reperfusion 24 h after ischemia (Fig. 4A). As basolateral uptake of organic anions is rate limiting, transepithelial secretion should be impaired at least to the same. PAH net secretion (PNS) is a measure of overall secretory transport by the proximal tubules of the kidney. Ischemia induced impairment of PNS is perfectly in the same range as impairment of PAH extraction ratio is (Fig. 4B). Moreover, PNS strongly correlates with PAH extraction ratio when single animals are considered (Fig. 4C). Thus, PNS is a function of PAH extraction ratio (and therefore of basolateral PAH uptake) even during reperfusion after renal ischemia and therefore PNS may be used to estimate development of PAH extraction ratio under these circumstances (and if renal vein is not cannulized). We, moreover, conclude that with respect to organic anion (PAH) handling altered expression of Oat1 and Oat3 is sufficient to explain the changes in renal function during reperfusion after ischemia. However, our data do certainly not exclude that the luminal exit step of transepithelial secretion is also affected.

The fact that PAH clearance does not reflect renal perfusion after renal ischemia and reperfusion was stated by Corrigan et al. (1999) and others (Dible et al. 1950; Eisenbach et al. 1974; Robinson et al. 1977; Finn and Chevalier 1979; Myers et al. 1984) since substantial time. However, this is the first study that gives mechanistic evidence explaining this particular phenomenon by impaired expression of the rate limiting basolateral organic anion transporters Oat1 and Oat3. Moreover, determination of PNS is easy when inulin clearance is determined in parallel to PAH clearance and allows approximation of renal excretion of PAH after ischemia if (1) renal extraction is set as 0.9 in untreated animals and (2) is then multiplied with the ratio of PNS in animals after ischemia and reperfusion to PNS in untreated animals. Based on these particular extraction ratios the respective PAH concentration in the renal vein can be calculated and an estimate for extraction corrected PAH clearance can be given (see equations in methods section). Even though this is an estimation the values will reflect RPF to a much better extent as uncorrected PAH clearance does after renal ischemia and reperfusion. This is even possible in a retrospective manner if inulin clearance was determined together with PAH clearance.

## Conflict of Interest

None of the authors has competing interests with respect to any issue of the respective study.
